# DigChem: Identification of disease-gene-chemical relationships from Medline abstracts

**DOI:** 10.1371/journal.pcbi.1007022

**Published:** 2019-05-15

**Authors:** Jeongkyun Kim, Jung-jae Kim, Hyunju Lee

**Affiliations:** 1 Gwangju Institute of Science and Technology, School of Electrical Engineering and Computer Science, Gwangju, Korea; 2 Institute for Infocomm Research, A-STAR, 138632, Singapore; University of Toronto, CANADA

## Abstract

Chemicals interact with genes in the process of disease development and treatment. Although much biomedical research has been performed to understand relationships among genes, chemicals, and diseases, which have been reported in biomedical articles in Medline, there are few studies that extract disease–gene–chemical relationships from biomedical literature at a PubMed scale. In this study, we propose a deep learning model based on bidirectional long short-term memory to identify the evidence sentences of relationships among genes, chemicals, and diseases from Medline abstracts. Then, we develop the search engine DigChem to enable disease–gene–chemical relationship searches for 35,124 genes, 56,382 chemicals, and 5,675 diseases. We show that the identified relationships are reliable by comparing them with manual curation and existing databases. DigChem is available at http://gcancer.org/digchem.

## Author summary

For understanding the role of chemicals in the molecular process of disease development and treatment, it is important to extract disease–gene–chemical relationships from the literature, that is, which gene and which chemical interact with each other for the development and treatment of which disease. Previous works extract binary relationships such as gene–chemical and disease–gene relationships from the literature and employ statistical measurements for deducing the triple relationship of disease–gene–chemical. Since the statistical measurements for inference often fail, we develop a deep learning model to identify the evidence sentences of disease–gene–chemical relationships from Medline abstracts. We show that the identified relationships are reliable, by comparing them with manually curated databases. We also provide a search engine called DigChem over the identified evidence sentences.

## Introduction

Much biomedical research has been performed to understand relationships among genes, chemicals, and diseases. These studies have been undertaken to reveal the molecular mechanisms underlying the activities of drugs, as the targets of many drugs remain unknown, despite the fact that they are known to be effective against diseases. Thus, extensive examinations of the mechanisms by which chemicals interact with genes in the development of diseases have been undertaken. Although these research results have rapidly accumulated in biomedical articles, databases or services providing disease–gene–chemical relationships directly extracted from articles at a PubMed scale are not available. Several existing databases such as DrugBank [1], the Comparative Toxicogenomics Database (CTD) [2] and the Therapeutic Target Database [3] provide manually-curated binary relationships, such as gene–chemical and chemical–disease associations. However, triplet relationships provided in these databases are indirectly generated based on inference over binary relationships.

Because manual curation is labor-intensive and costly, especially when considering the rapid growth of biomedical literature, several natural language processing methods have been proposed to automatically recognize relationships between entities. Text mining tools, including RELigator [4] and UET-CAM [5], have been developed to extract chemical–disease relationships. These systems participated in the BioCreative V Chemical Disease Relation (CDR) Task [6], achieving F-scores of 0.526 and 0.516, respectively. Xu and Wang [7] developed a pattern learning approach to extract drug–disease pairs from biomedical abstracts, achieving a precision of 0.904 and a recall of 0.131 when testing it with an in-house data set. Recently, several deep learning methods have been proposed to identify relationships among biomedical entities, showing better performance than previous machine learning approaches. DRMDA [8] proposed a microRNA-disease association prediction model using an auto-encoder that represents disease semantic similarity and miRNA functional similarity. Sahu et al. [9] developed a framework based on a convolutional neural network (CNN) for extracting relations among biomedical entities, achieving an F-score of 71.16% when applied to evaluation data provided by Informatics for Integrating Biology and the Bedside (i2b2) in 2010 as part of the i2b2/VA challenge [10]. Xu et al. [11] proposed a bidirectional long short-term memory (bi–LSTM) network-based method for extracting drug–drug interaction from biomedical literature, achieving an F-score of 71.15% on SemEval 2013 task 9.2 data set [12]. Li et al. [13] developed a bi–LSTM-based framework using a dynamic extended tree for extracting biomedical events among biotope and bacteria from biomedical literature, which achieved 57.14% of F-score in the BioNLP’16 Shared Task on Bacteria Biotope task [14]. However, these tools identified relationships between two entities only, not triplet relationships.

In this study, we propose a deep learning method to identify pairs of sentences that explicitly express the disease–gene–chemical relationships based on bi–LSTM. Here, we assume two sentences together represent a triplet relationship: one sentence representing gene–chemical relationship and another sentence representing a disease related to the gene–chemical relationship. If the three elements appear in the same sentence, the sentence is duplicated into two identical sentences. Then, we apply the proposed method to sentence pairs from Medline abstracts, and build a disease–gene–chemical relationship search engine (DigChem) to retrieve the triplet relationships extracted from PubMed.

## Materials and methods

### Relationship classification model

We develop a relationship classification model to recognize a chemical-gene-disease relationship based on sentences extracted from literature. For this task, we generate a gold standard data set containing positive and negative sentences, and then develop a deep learning model to classify positive and negative sentences.

#### Gold standard evidence sentences

We define ‘positive evidence sentences’ as those describing the direct or indirect interaction of a chemical with a gene, and subsequently claiming that the chemical and the gene are related to a disease. On the other hand, we define ‘negative evidence sentences’ as those that do not describe triplet relationship, despite the fact that they contain chemical, gene, and disease names. Because sentences with three entities are infrequently found in Medline abstracts, we collect evidence consisting of two sentences: a gene–chemical sentence and a disease sentence. A chemical term and a gene term appear in the gene–chemical sentence, and a disease term appears either in the same sentence or in another sentence describing a disease. If the disease term appears in the gene-chemical sentence, the disease sentence is the same as the gene–chemical sentence. Examples of positive and negative sentence pairs are shown below.

- Positive sentence pairs for gene *BNP*, chemical *SUN*, and disease *renal cell carcinoma* (PMID: 24984876).
  - Sentence 1: “At the protein level, Western blot analysis showed that *SUN* increased *BNP* and b-MHC, while it inhibited a-MHC protein levels in a concentration-dependent manner.”
  - Sentence 2: “Sunitinib (*SUN*) is a multi-targeted tyrosine kinase inhibitor used for the treatment of gastrointestinal stromal tumors and *renal cell carcinoma*.”
- Negative sentence pairs for gene *ACE*, chemical *hydralazine*, and disease *glomerulosclerosis* (PMID: 25143333).
  - Sentence 1: “CONCLUSION: The results show following an abrupt decline in podocyte number, the initiation of *ACE*-inhibition but not *hydralazine*, was accompanied by higher podocyte number in the absence of proliferation.”
  - Sentence 2: “OBJECTIVE: The objective of this article is to test the effects of angiotensin-converting enzyme (*ACE*)-inhibition on glomerular epithelial cell number in an inducible experimental model of focal segmental *glomerulosclerosis* (FSGS).”

To construct gold standard sentences, we randomly select sentence pairs from those Medline abstracts that contain chemical, gene, and disease mentions, and then manually evaluate them as positive or negative sentences, resulting in 1000 positive and 1000 negative triplets from 1,984 gene–chemical sentences and 1,900 disease sentences. For 500 positive and 500 negative triplets out of the 2,000 triplets, their gene–chemical sentences and disease sentences are identical, which means that the three mentions of each of those triples are from the same sentence. Also, if a sentence has multiple mentions of gene and of chemical, each pair of gene and chemical is extracted to form either positive or negative triplet with a disease mention. In fact, six gene–chemical sentences in the corpus have multiple gene–chemical pairs, and 35 disease sentences are related to the multiple gene–chemical pairs.

In addition, we consider two other configurations of two sentences as well: a gene–disease sentence and a chemical sentence, and a chemical–disease sentence and a gene sentence. We examine frequencies of triplets with the three combinations in randomly selected 100 abstracts, and observe 1,058 gene–chemical and disease pairs, 694 chemical–disease and gene pairs, and 515 gene–disease and chemical pairs. Furthermore, we compare the three configurations by evaluating our approach with the 500 positive and 500 negative triplets whose gene, chemical, and disease mentions are from the same sentence, and found that they do not show any significant difference in performance (see the Results section for details). As such, because triplets from a gene–chemical sentence and a disease sentence are most frequently observed, we use this configuration in this study.

#### Word embedding features

**Fig 1:**
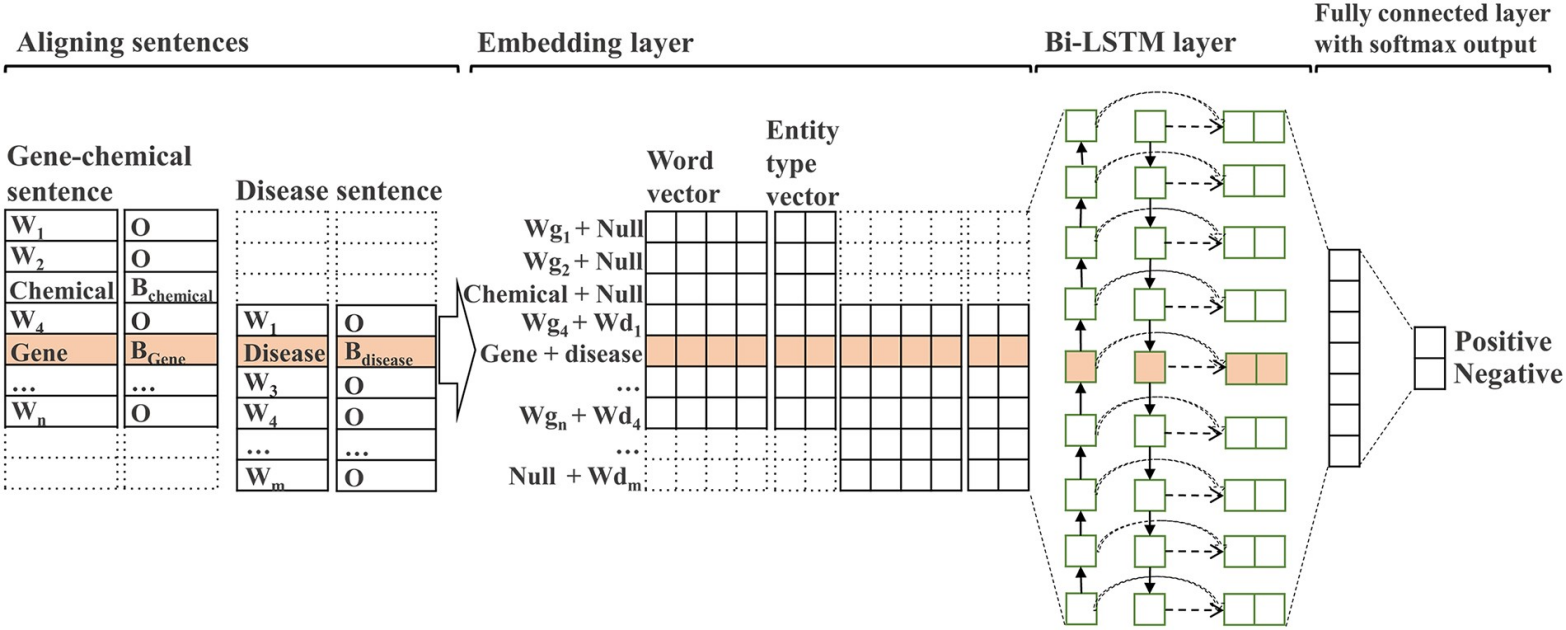
Architecture of the relationship classification model.

In our proposed model, we represent each word in a sentence using word embeddings. Word embeddings can be used to express semantics of words and information about sentences, such as parts of speech, phrases, and entity types. Here, we use two embedding features: word representation vectors and entity type representation vectors (Fig 1). Word representation vectors are usually pre-trained using a huge, unannotated data set in which words with similar semantics have high vector similarities. We use word vectors with a vector size of 200 that are pre-trained by applying Word2Vec [15] to MEDLINE data, which provides word vectors specialized for biomedical articles. Entity type representation vectors represent the entity types of words obtained from NER tools. Because we have three entity types (chemicals, genes, and diseases) in sentences, the entities are tagged with a BIO format (7 tags in total), where B, I, and O represent beginning, inside, and outside of an entity, respectively. The representation vector for each of the 7 tags is randomly initialized with a vector size of 20. By concatenating the two vectors, the size of a vector for a word becomes 220.

#### Bi–LSTM

Among several neural network models, recurrent neural networks (RNNs) may be most suitable for modeling sequential data, because the previous hidden state together with the current input is used to represent the current hidden state. One drawback of the RNN model is gradient vanishing. In the training step of RNN, back-propagated gradients either grow or shrink. Therefore, after many steps or in sequences which are too long, gradients explode or vanish. Long short-term memory (LSTM) was first proposed by Hochreiter and Schmidhuber [16] to overcome the gradient vanishing problem of RNNs. They present a mechanism called adaptive gating, which decides the degree to which an LSTM unit keeps the previous state and memorizes the features extracted from the current data input. An LSTM unit has three multiplicative gates that control the proportions of information to be forgotten or passed on to the next time step.

Furthermore, a sequence modeling task may benefit from access to both past and future context. Schuster and Paliwal [17] proposed bi-LSTM, which extends unidirectional LSTM by introducing a second hidden unit, where the hidden-to-hidden connections inside the two units flow in reverse orders. Thus, the two hidden states of bi-LSTM can capture both past and future information. The two hidden states are then concatenated to form the final output. We use bi-LSTM in the relationship classification model to consider the past and future context for identifying the relationships. Fig 1 illustrates the architecture of the proposed relationship classification model.

Our goal is to collect pairs of two sentences, one with gene-chemical relationship and another with a disease related to the gene-chemical relationship. While conventional bi-LSTMs take a single sequence (e.g. a sentence) as an input, our method takes as input a pair of two sentences, one with both a gene name and a chemical name and another with a disease name, and identifies if the former sentence expresses the supposed relation between the gene and the chemical and the latter sentence the relationship between the disease and the gene-chemical relationship. We thus propose to concatenate the two sentences in parallel, aligned at the positions of the gene and disease mentions. Words in the sentences are represented as the embedding vector as described in ‘Word embedding features’ section. As a result, the input data of our proposed model is a 2-dimensional array, where the number of rows is twice the vector size of word embeddings and that of columns is the maximum length of two aligned candidate sentences.

Our model has two parts: a bi-LSTM layer and a fully-connected layer. It concatenates the hidden states of the two LSTMs of the bi-LSTM for each word and passes the sequence of the concatenated hidden states as the input of the fully-connected layer which generates softmax output. The output layer consists of positive and negative classes with probabilities that are used to rank sentences during the search engine’s searching process.

### Extracting relationships from Medline abstracts

To extract disease–gene–chemical relationships at a PubMed scale, we perform three tasks. First, we annotate gene, chemical, and disease names by applying named entity recognition (NER) tools to Medline abstracts. Next, we apply the proposed bi-LSTM model to extract the triplet relationship. Finally, we employ a post-processing step to remove false positives from the predicted relationships.

#### Named entity recognition

To extract mentions of gene, chemical, and disease from Medline abstracts, three NER tools are used: GNormPlus [18] for gene names, tmChem [19] for chemical names, and DNorm [20] for disease names. We obtain the NER results from PubTator [21], which is freely accessible at https://www.ncbi.nlm.nih.gov/CBBresearch/Lu/Demo/PubTator/.

GNormPlus analysis consists of two steps: mention recognition and concept normalization. A conditional random fields (CRF) model is used to recognize gene mention, and a statistical inference network model with two matching strategies is used to map gene mentions to specific concepts. The recognized gene mentions are normalized to Entrez gene concepts. GNormPlus [18] achieved a precision of 87.3%, a recall of 86.4%, and an F-measure of 86.7% on the BioCreative II gene normalization test set.

Chemical names and their identifiers are identified in the biomedical literature using tmChem [19]. It participated in the CHEMDNER task [22], and achieved a precision of 89.1%, a recall of 85.8%, and an F-measure of 87.4%, which was the highest F-measure in the chemical entity mention recognition subtask of CHEMDNER [22]. tmChem uses the CRF model and maps chemical entities to Medical Subject Headings (MeSH) and Chemical Entities of Biological Interest (ChEBI) identifiers.

Disease mentions are identified and normalized using DNorm [20], which achieved a precision of 80.3%, a recall of 76.3%, and an F-measure of 78.2% using the NCBI disease corpus. DNorm recognizes disease mentions with BANNER [23], and normalizes them to MEDIC concepts using a pairwise learning model.

#### Post-processing step

Because predicted positive relationships may contain false positive sentence pairs, we apply a post-processing step to screen the false positive sentence pairs. We observed that there were four majority false positive cases: i) incorrectly recognized gene, chemical, and disease mentions from NER tools, ii) an experimental procedure or purpose of research, iii) a sentence pair that explicitly expresses no relation among gene, chemical, and protein, and iv) diseases in the disease sentence being unrelated to genes and chemicals in the gene–chemical sentences. Examples of these four cases are shown in S1 Table.

Based on these observations, we construct five rules to filter false positive sentences: i) a sentence pair is filtered out when recognized mentions are not contained in synonyms of entities in dictionaries after the recognized mentions are normalized into entity names, ii) a sentence pair is filtered out if any mention is recognized as more than one entity type. For example, if one mention is recognized as a gene and a chemical by the gene NER tool and the chemical NER tool, respectively, at the same time, iii) a sentence pair is filtered out if it contains hyponyms of ‘study’ from WordNet, because it may express a purpose of research, iv) a sentence pair is filtered out if it contains negation keywords such as ‘not’ and ‘never’, and v) when gene name and chemical name are connected by a conjunction in the dependency parse tree, this sentence is filtered out because genes and chemicals do not interact with each other in most of these cases.

#### Indexing and searching

We construct a search engine to retrieve disease–gene–chemical relationships extracted from Medline abstracts. The DigChem search engine consists of indexing and searching processes. In the indexing process, an inverted index is built to efficiently access the positive sentences that are obtained by the proposed model followed by the post-processing step. The inverted index contains information about a gene, a chemical, and a disease type in each sentence pair.

In the searching process, indexed evidence sentence pairs are searched for an input query. The input query consists of gene, chemical, and/or disease, and the inverted index is used to return sentence pairs that express the relationship among them. The query does not necessarily require gene term and chemical term information. When the query does not contain a gene term or a chemical term, the system returns all genes or all chemicals related to the given disease type. Retrieved evidence sentence pairs are sorted by scores obtained from the relationship classification model. The Apache Lucene search engine library was used to build the indexing and searching processes.

## Results and discussion

### Performance of the relationship classification model

**Fig 2:**
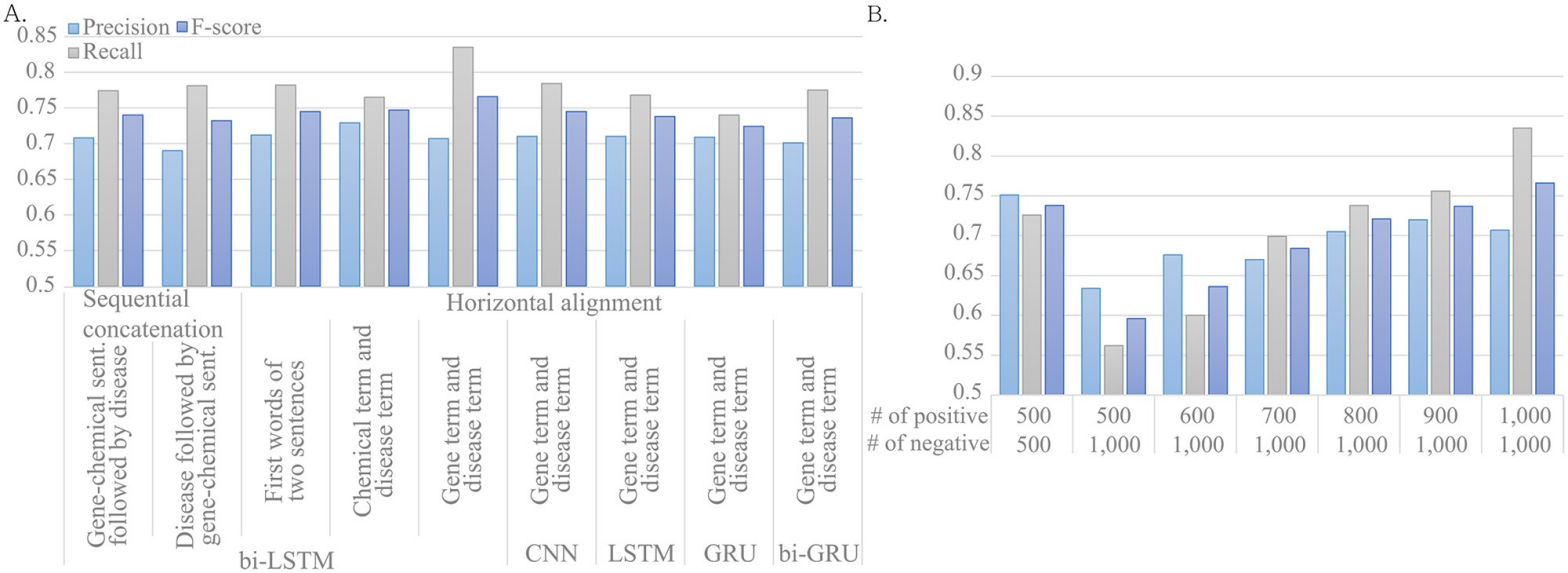
Performances of relationship classification models. (A) Performances of relationship classification models for different neural networks and different ways of combining the two sentences of input. (B) Performances of the proposed model for different amounts of positive and negative samples.

Performance of the proposed bi-LSTM model was measured by 10-fold cross-validation using the gold standard data. We tested several values for the following four hyperparameters: the number of hidden units of LSTM, the learning rate, the size of the fully connected layer, and the size of mini-batches. Fig 2A and S2 Table show the performance of the model with the following best hyperparameters; the number of hidden units of LSTM units: 100, the learning rate: 0.80, the size of the fully connected layer: 1,000, and the size of mini-batches: 200. The results with other hyperparameters are shown in S3 Table. We also compared the performance of the proposed model with other neural network-based models including CNN, gated recurrent unit (GRU), LSTM, and bi-directional GRU with the same hyperparameters as the bi-LSTM model. Note that the architectures of other neural networks are similar to the proposed model, with only the neural network layer changed, as seen in Fig 1. As shown in Fig 2A, the bi-LSTM model achieved an F-measure of 76.6%, outperforming the other neural network models.

In the proposed classification model, we aligned the position of the gene mention in the gene-chemical sentence with that of the disease mention in the associated disease sentence. In fact, we compared different ways of combining the two sentences of input, including sequential concatenation (the number of rows is identical to the vector size of word embeddings) and concatenation in parallel (or horizontal alignment) at different anchor positions (e.g. the first words of the two sentences, chemical and disease mentions), with the proposed way of horizontal alignment at the positions of the gene and disease mentions. We compared these combination ways by evaluating them with the neural network models. Fig 2A and S2 Table summarize the evaluation results. The proposed alignment shows the best performance, possibly because the explicit alignment of two potentially related entities may guide the bi-LSTM to focus on their relation. In particular, the proposed alignment improves the performance of the bi-LSTM from 0.740 to 0.766 of F-score.

For 500 positive and 500 negative triplets in the gold-standard data, gene, chemical, and disease are in the same sentence. Thus, using these 1000 triplets, we compared the performance of the three configurations, gene–chemical and disease pairs, chemical–disease and gene pairs, and gene–disease and chemical pairs, and obtained F-measures of 0.736, 0.733, and 0.735, respectively. These results do not show any significant difference in performance among the three configurations.

At the PubMed scale, the number of negative sentence pairs might be considerably larger than that of positive sentence pairs. Thus, to examine the effect of different ratios of positive and negatives samples for training the proposed method, we measured the performance of the proposed model using the several different sets of positive and negative gold standard samples (Fig 2B and S4 Table). In the results, training with 500 positive and 500 negative sentence pairs achieved an F-measure of 0.738, while training with 500 positive and 1,000 negative sentence pairs achieved an F-measure of 0.596. It shows that the proposed model may be well trained with the similar amount of positive and negative sentence pairs.

### Post-processing

After we applied the proposed bi-LSTM model to all Medline abstracts, we randomly selected and analyzed 400 positive sentence pairs to measure the performance of predicted relationships. As shown in Table 1, 35.8% of the sentence pairs were false positives. Incorrect gene-chemical relationship (19% of the positive sentence pairs) was the most frequent case, followed by NER errors (9.5%). This precision was lower than the precision measured using the gold standard evidence sentences in Fig 2A, because the NER errors were combined with the relationship classification error.

**Table 1:** Results of post-processing.

		True positive sentences	NER error	Purpose or process of research	Incorrect gene-chemical relationship	Incorrect disease relationship	Total
For generating post-processing rules	Before	257	38	14	76	15	400
	After	239	29	2	22	7	299
For testing	Filtered sentences	27	26	4	27	15	99
	Non-filtered sentences	68	8	0	13	12	101

For filtering the false positive sentence pairs, we constructed a rule-based post-processing step described in the Methods section, and filtered 101 sentence pairs. While the post-processing step was useful for filtering incorrect relationships, it was less effective in case of NER errors. Out of the 38 NER errors, 29 NER errors still remained after post-processing. The breakdown of the NER errors according to entity types (gene, chemical, and disease) is shown in S5 Table. The post-processing rules were most effective to reduce disease NER errors and thus filtered out more sentences with disease NER errors than those with gene and chemical NER errors, while they were not so effective for gene NER errors.

To show the effect of the post-processing step, we compared the final results of the proposed method with those that are filtered, thus discarded, by the post-processing step as follows: we randomly selected 99 sentence pairs that are filtered by the rules and 101 sentence pairs that are not filtered by the rules. While 67.3% (68 / 101) of the non-filtered sentences are true positive, only 27.3% (27 / 99) of the filtered sentences are true positive (Table 1). This result suggests that the post-processing rules are effective to filter negative sentences although some positive sentences are incorrectly filtered together.

### Statistics of classification results

From Medline abstracts, 11,648,261 sentence pairs were retrieved which contained gene, chemical, and disease names. After applying the proposed model and the post-processing step to these sentence pairs, 2,136,416 were recognized as positive evidence sentence pairs, and 5,675 diseases, 35,124 genes, and 56,382 chemicals were indexed. Unique gene–chemical–disease relationships were found in 943,985 triplets. Table 2 shows the ten most frequently identified relationships. The relationship of insulin, glucose, and diabetes mellitus appeared most frequently. The evidence sentence pairs of the ten most frequently identified relationships are shown in S6 Table. One hundred and sixty seven relationships were recognized in more than 100 sentence pairs, while 525,705 relationships, approximately half of the unique positive relationships, were retrieved only once.

**Table 2:** The ten most frequently recognized relationships.

Gene ID	Gene symbol	Chemical ID	Chemical identifier	Disease ID	Disease identifier
3630	INS	D005947	Glucose	D003920	Diabetes Mellitus
25	ABL1	C097613	imatinib	D015464	Leukemia, Myelogenous, Chronic, BCR-ABL Positive
3091	HIF1A	D010100	Oxygen	D000860	Hypoxia
367	AR	CHEBI:50113	androgen	D011471	Prostatic Neoplasms
2475	MTOR	D020123	Sirolimus	D009369	Neoplasms
1956	EGFR	C419708	gefitinib	D009369	Neoplasms
3630	INS	D005947	Glucose	D007333	Insulin Resistance
2099	ESR1	D004967	Estrogens	D001943	Breast Neoplasms
3064	HTT	C097188	polyglutamine	D006816	Huntington Disease
673	BRAF	C551177	vemurafenib	D008545	Melanoma

**Fig 3:**
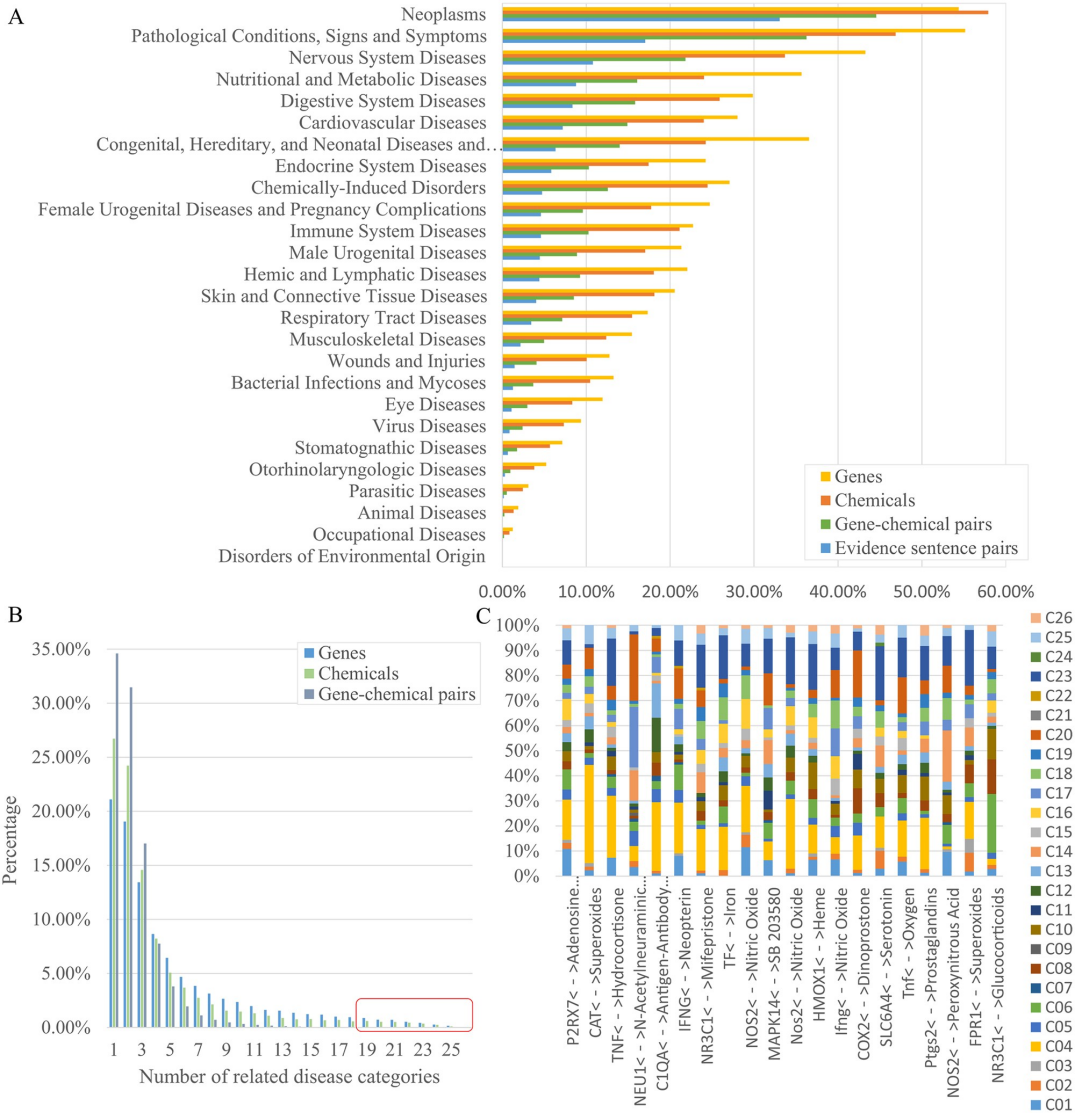
Statistics of DigChem. (A) Distribution of the number of related genes, chemicals, gene-chemical pairs, and the number of evidence sentence pairs for general disease categories. (B) The number of related diseases for genes, chemicals, and gene-chemical pairs. (C) For the gene-chemical pairs related to more than 19 MeSH disease categories, the spectrum of related diseases is shown. C01–C26 represent the disease category identifiers used in MeSH.

Fig 3A shows the numbers of evidence sentence pairs and retrieved genes, chemicals, and gene-chemical pairs for the 26 most general disease categories in MeSH. Gene–chemical relationships were most frequently identified in neoplasms, followed by pathological conditions, signs and symptoms. Interestingly, the endocrine system diseases and chemically-induced disorders categories contain a relatively larger number of evidence sentence pairs (ranked 8th and 9th, respectively, among 26 disease categories) when compared to the number of articles related to these diseases, which are ranked 16th and 20th, respectively. We investigated which genes, chemicals, and gene–chemical pairs were involved in multiple disease types across the 26 disease categories (Fig 3B). About 65% of the gene–chemical pairs are related to only one or two disease categories, while 20 gene–chemical pairs, such as the TNF–hydrocortisone and TF–iron pairs, are related to more than 19 disease categories. Fig 3C illustrates the spectrum of related disease categories for the 20 gene–chemical pairs.

### Comparison with existing databases

To assess the reliability of DigChem, we collected triplet relationships from DrugBank (version 4.0) [1] and CTD [2], and compared them with triplets retrieved by DigChem. DrugBank is a well-known database containing biochemical and pharmacological drug information. DrugBank services the mechanisms of drugs and manually-curated drug’s target genes. Disease–drug relationships are described in pharmacological information related to drugs in DrugBank. Because disease and pharmacological information is described in unstructured text, we use a disease NER tool, DNorm, to find disease terms. We then construct triplet relationships with serviced drug–gene relationships, resulting in 14,377 triplets. CTD provides manually-curated interactions of chemical–gene, chemical–disease, and gene–disease pairs from the literature. These curated relationships were computationally integrated to generate candidate gene–chemical–disease associations by inferring genes commonly linked to chemicals and diseases. As a result, CTD provides a total of 4,758,144 indirect triplet relationships.

**Fig 4:**
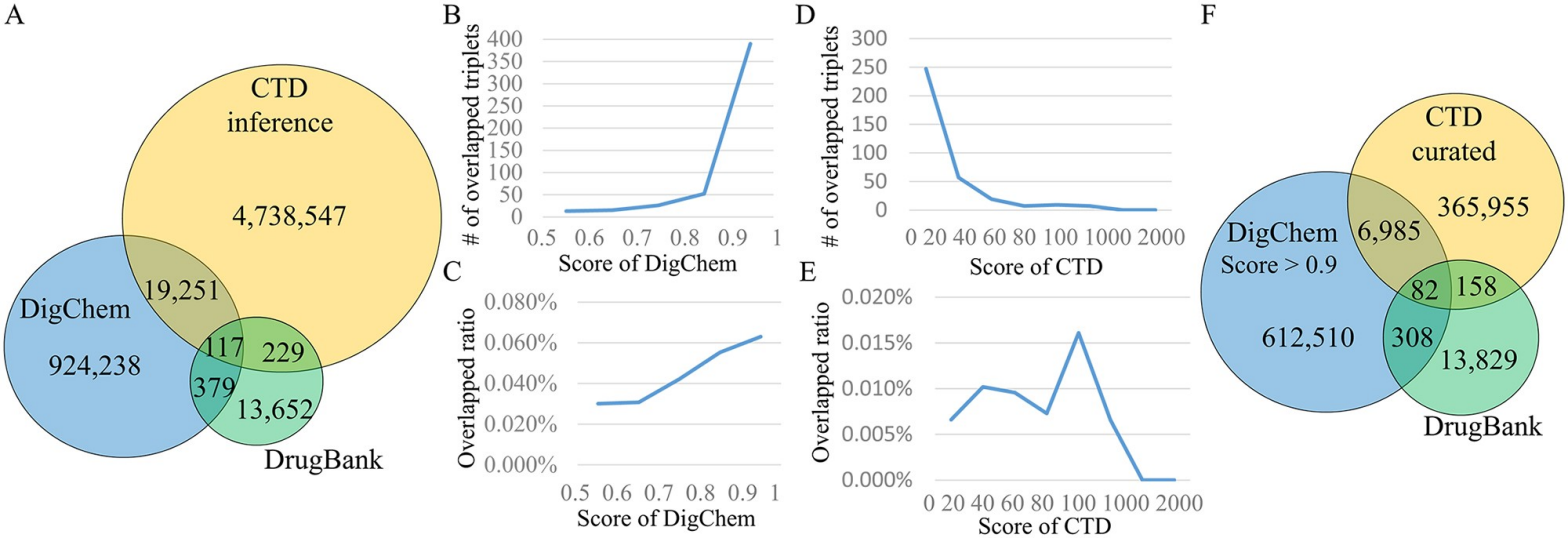
Comparison results with CTD and DrugBank. (A) The number of common triplet relationships in CTD, DrugBank, and DigChem. (B) The number of overlapping triplet relationships identified by DigChem and DrugBank. (C) Overlap ratio of triplets identified by DigChem and DrugBank. (D) The number of overlapping triplet relationships by CTD and DrugBank. (E) Overlap ratio of triplets by CTD and DrugBank. (F) The number of common triplet relationships in the CTD curated set, DrugBank, and the relationships which scored over 0.9 in DigChem.

Considering DrugBank as a widely-accepted dataset, we compared its triplet overlap with triplets from DigChem and CTD. Of 943,985 unique triplet relationships identified by DigChem, 496 relationships (3% of DrugBank) were shared with DrugBank (Fig 4A). Although this overlap was small, the overlap between DrugBank and CTD was even smaller, as 4,758,144 relationships in CTD covered only 2.4% of those found in DrugBank. In addition, we checked the literature references of the drug targets described in DrugBank, but not identified by DigChem. In some cases, these reference articles did not contain gene, chemical, and disease names in abstracts. In other cases, although triplets were described in abstracts, gene and chemical names were written in separate sentences, and in these cases, DigChem could not identify their relationship. Among 4,758,144 relationships in CTD, 19,368 relationships (0.4% of CTD) were shared with DigChem (Fig 4A).

In addition, we verified statistical significance of overlapping triplets between DigChem and other databases, CTD and DrugBank. S1 Fig shows the binomial distribution of overlapping probabilities of randomly generated triplets. Because the number of triplets in DigChem is 943,985, we generated the distribution of the number of overlapping triplets between 4 billion and 943,985 out of all possible triplets for CTD, and that between 14,377 and 943,985 out of all possible triplets for DrugBank. The binomial test achieved p-values < 2.2e-16. Moreover, we checked triplets of DigChem and CTD, which contain chemicals in DrugBank triplets. We collected 161,306 triplets from DigChem and 913,470 triplets from CTD, where 7,866 triplets overlap between DigChem and CTD (S1 Fig). In this case, p-values were also less than 0.05.

We also investigated whether relationships predicted with higher scores by CTD and DigChem are more likely to overlap with those predicted by DrugBank. We calculated the number of overlaps and overlap ratios for each score range of DigChem (Fig 4B and 4C), and of CTD (Fig 4D and 4E). The relationships having higher scores in DigChem were more likely to be described in DrugBank, while the relationships in CTD did not show any correlation between inference scores by CTD and overlap ratios with DrugBank. This shows that the relationships with higher scores predicted by the proposed model are more reliable than those with lower scores.

We further investigated ‘reliable’ triplets from DigChem and from CTD. For this purpose, we selected 619,885 triplets having scores > 0.9 in DigChem, referred to as high-score DigChem triplets. Among the 4,758,144 indirectly inferred triplets of CTD, based on common genes between chemical–gene and gene–disease relationships, we extracted a subset of 373,180 triplets, referred to as curated CTD triplets, by connecting all three gene, chemical, and disease elements that are common in three binary relationships: gene–chemical, chemical–disease, and gene–disease relationships (Fig 4F). Though the overlap ratios of these subsets with DrugBank are similar, with curated CTD triplet overlap being 0.064% (240 / 373,180) and high-score DigChem overlap of 0.063% (390 / 619,885), DigChem provides a higher number of ‘reliable’ triplets than CTD. S7 Table presents 50 example evidence sentence pairs, which are identified by DigChem but missed by the existing databases.

### Case studies of four diseases

We analyzed triplet relationships related to four diseases: Alzheimer’s disease, breast cancer, hypertension, and prostate cancer. The term “breast cancer” was found in 47,580 sentence pairs, and each of the other three diseases were found in approximately 20,000 sentence pairs. The number of unique relationships among genes, chemicals, and diseases ranged from 6,872 to 15,488 (Table 3).

**Table 3:** The numbers of sentence pairs, genes, chemicals, and relationships for four diseases by DigChem, and the numbers of triple relationships from CTD, DrugBank, and WDD for four diseases.

	DigChem				Triplets by CTD	Triplets by DrugBank	Triplets by WDD
	Sentence pairs	Related genes	Related chemicals	Unique triplets			
Alzheimer’s disease	17,804	1,929	2,440	6,872	21,230	183	45,073
Breast cancer	47,580	3,476	4,307	15,488	73,627	105	78,654
Hypertension	21,021	2,218	2,388	8,324	44,661	389	42,709
Prostate cancer	28,327	2,168	2,618	8,440	72,379	36	67,690

**Fig 5:**
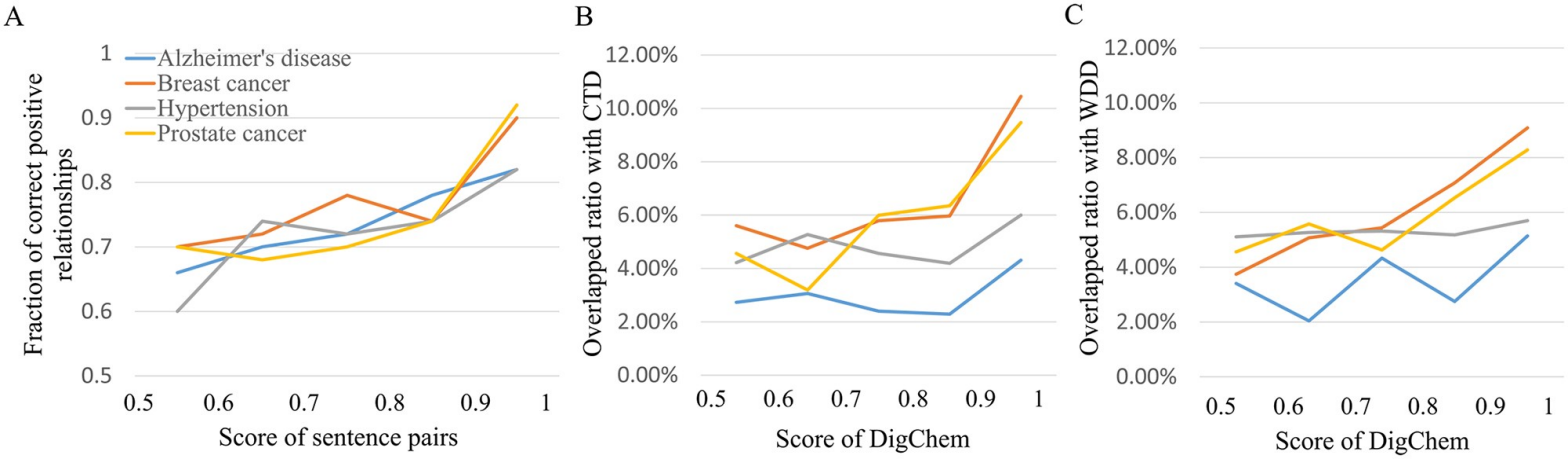
Case studies with four diseases; Alzheimer’s disease, breast cancer, hypertension, and prostate cancer. (A) The fractions of correct positive relationships according to the prediction scores. (B) The overlapping ratio with CTD according to the prediction scores by DigChem. (C) The overlapping ratio with WDD according to the prediction scores by DigChem.

To evaluate the retrieved triplet relationships, we randomly selected 50 sentence pairs for each of five score ranges (from 0.5–0.6 to 0.9–1.0) and manually checked whether they contained positive relationships. Note that pairs with scores lower than 0.5 were classified as negative sentence pairs. Fig 5A shows that the sentence pairs with higher scores are mostly more reliable. For the example cases of breast and prostate cancers, more than 90% of relationships with scores larger than 0.9 were correct, while only 70% of those with lower scores (0.5–0.6) were correctly identified as positive relationships.

We also compared the retrieved triplet relationships with existing databases: CTD, DrugBank, and IBM Watson for Drug Discovery (WDD). WDD is a machine learning solution that analyzes drug information to help researchers identify novel drug targets and indications. WDD services relationships among genes, chemicals, and diseases extracted from literature, and contains gene–disease, chemical–gene, and chemical–disease relationships. It does not, however, contain triplet relationships. We therefore generated triplets from WDD by connecting common genes, diseases, and chemicals in three binary relationships. Table 3 shows triplets collected for four diseases from DigChem, CTD, DrugBank, and WDD. Note that in this comparison we used the curated-CTD triplets detailed in the section describing comparisons with existing databases.

**Fig 6:**
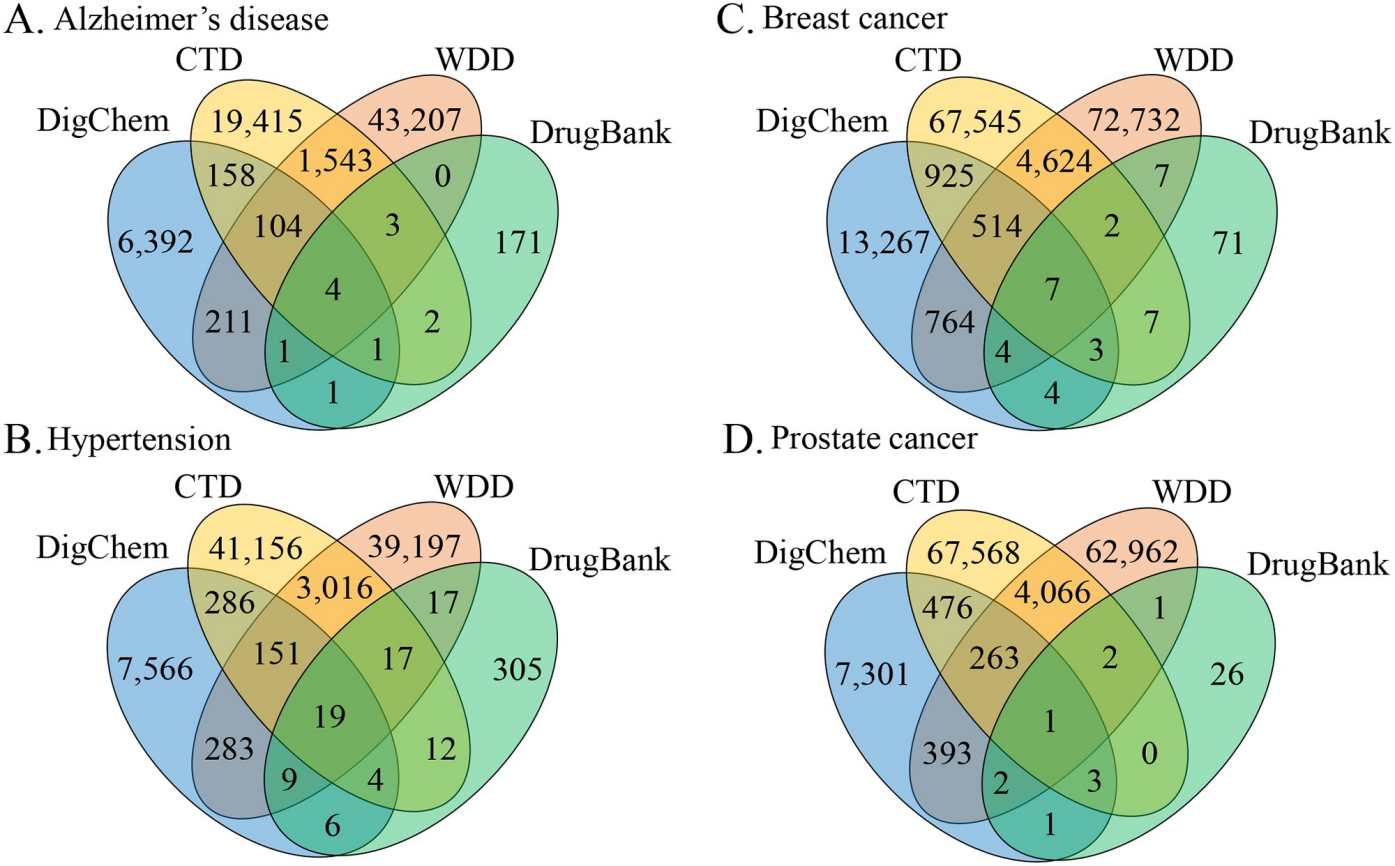
Overlapping triple relationships among, DrugBank, CTD, WDD and DigChem. (A) Alzheimer’s disease. (B) Hypertension. (C) Breast cancer. (D) Prostate cancer.

Fig 5B and 5C show overlapping ratios of DigChem with CTD and WDD, segmented by the prediction scores of DigChem. In breast and prostate cancers, triplet relationships from DigChem having higher scores shared a greater degree of overlap with CTD and WDD. In Alzheimer’s disease and hypertension, triplets having scores > 0.9 mostly overlapped with CTD and WDD. Furthermore, when we compared overlapping triplets among the four databases, overlap ratios of up to 10% were found (Fig 6). These results indicate that currently existing databases are complementary to each other.

Moreover, to illustrate how the extracted triplets can be used in disease research, we used an Alzheimer’s disease pathway from KEGG [24] (https://www.genome.jp/kegg-bin/show_pathway?hsa05010). DigChem retrieved a total of 6,872 unique triplets, and we found that 19 genes in the pathway have relationships in DigChem (S2 Fig). Among these, 15 genes are related to FDA-approved drugs. DigChem retrieved a total of 6,872 unique triplets, and we found that 19 genes in the pathway have relationships in DigChem (S2 Fig). Among these, 15 genes are related to FDA-approved drugs. In addition, 10 genes in the pathway have triplet relationships in CTD, and they are all related to FDA-approved drugs. Among the genes identified in CTD and DigChem, 6 genes are common. S8 Table presents 62 evidence sentence pairs for the triplets of the 19 genes. These 19 genes might be good candidates for further research for the treatment of Alzheimer’s disease.

### Web interface of DigChem

We developed a web interface to provide a service which may be used to search for relationships among genes, chemicals, and diseases. A DigChem query consists of the following three elements: 1) a list of disease(s), 2) a list of gene(s), and 3) a list of chemical(s). If a query does not contain any gene name, all genes are considered during relationship search. Similarly, if no chemical name (or disease description) is given, all chemicals (or all diseases) are considered during the search. Synonyms of disease terms and chemical names collected from the MeSH database [25] are searched together with the input terms. Search results consist of a list of triplets and the evidence sentence pairs supporting their relationship. Users can download search results as a tab-delimited file. Moreover, users can upload manually curated evidence sentences.

## Supporting information

S1 Fig Binomial distribution of overlapping probability between other databases and DigChem. (PDF)

S2 Fig Triplet relationship appearing in the Alzheimer’s disease pathway. (PDF)

S1 Table Example sentence pairs of post-processing cases. (XLSX)

S2 Table Performance of relationship classification models. (XLSX)

S3 Table Performance of hyper-parameter tuning. (XLSX)

S4 Table Performance of the proposed model for different amount of positive and negative samples. (XLSX)

S5 Table Breakdown of NER errors of post-processing. (XLSX)

S6 Table Evidence sentence pairs of the ten most frequently recognized relationships. (XLSX)

S7 Table Evidence sentence pairs that are identified by DigChem but missed by existing databases. (XLSX)

S8 Table Evidence sentence pairs about genes from the KEGG pathway (Alzheimer’s disease). (XLSX)
