# Supplementary figures and images for: DigChem: Identification of disease-gene-chemical relationships from Medline abstracts
Source: PLoS Comput Biol. 2019 May 15;15(5):e1007022. doi: 10.1371/journal.pcbi.1007022 (PMC6519793; doi:10.1371/journal.pcbi.1007022)

A.

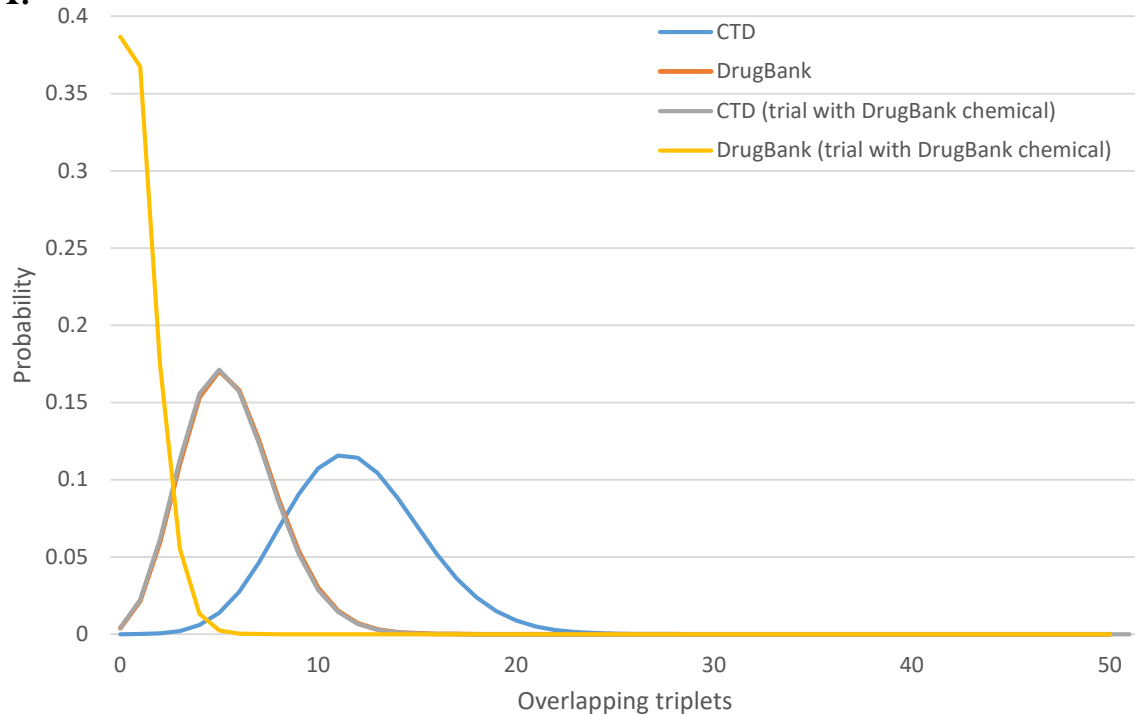

B.

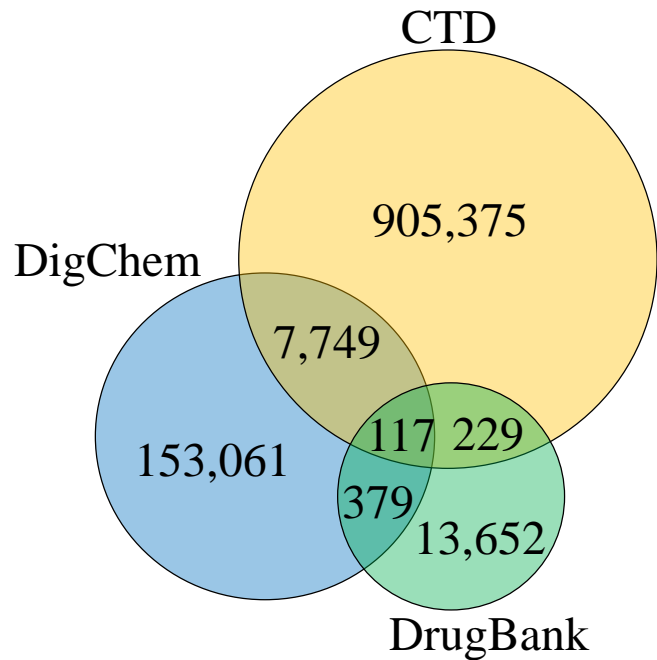

Supplement: S1 Fig — (PDF) [file pcbi.1007022.s001.pdf]

# ALZHEIMER'S DISEASE

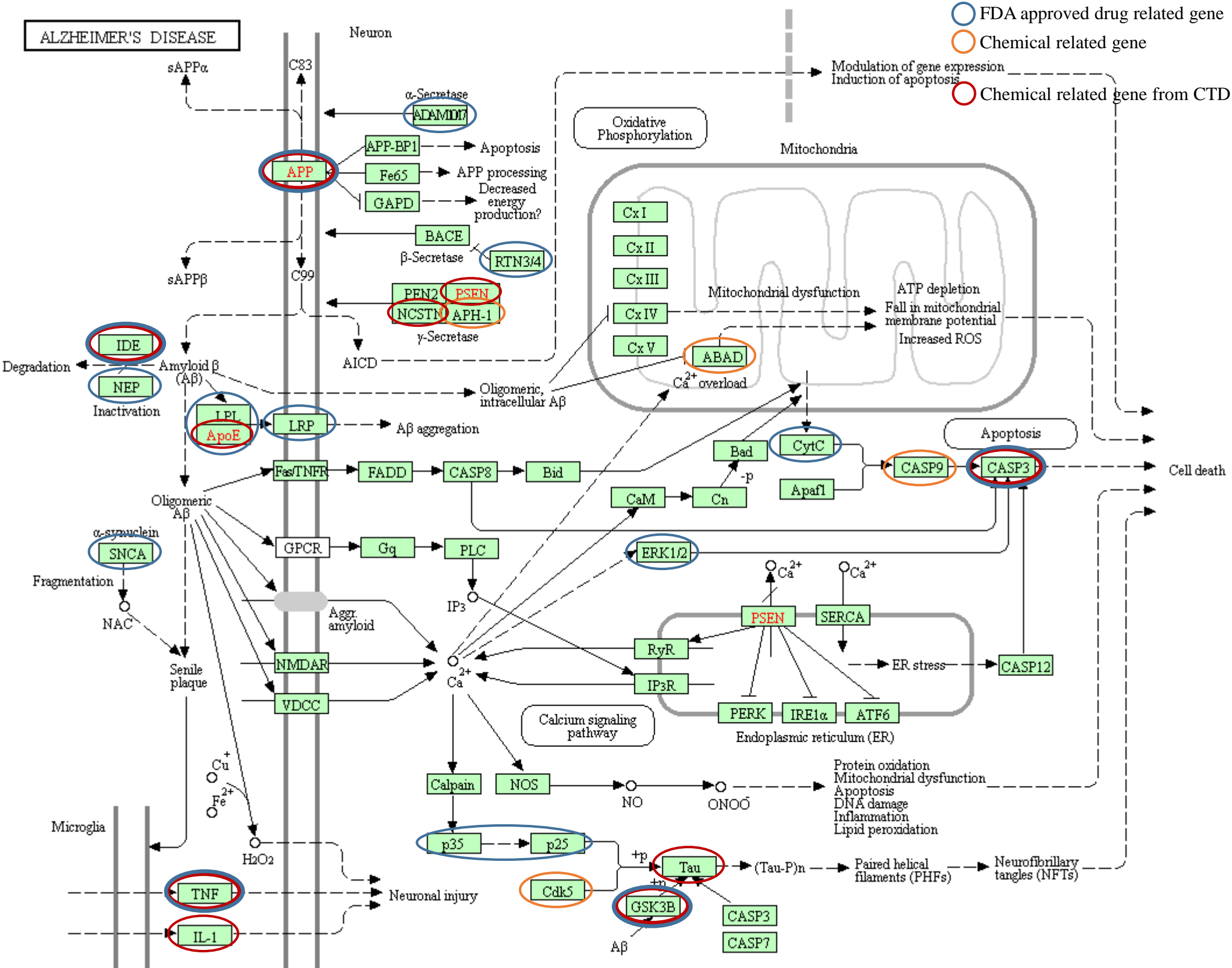

Supplement: S2 Fig — (PDF) [file pcbi.1007022.s002.pdf]
